# Boger nanofluid: significance of Coriolis and Lorentz forces on dynamics of rotating fluid subject to suction/injection via finite element simulation

**DOI:** 10.1038/s41598-022-05487-2

**Published:** 2022-01-31

**Authors:** Bagh Ali, Imran Siddique, Sajjad Hussain, Liaqat Ali, Dumitru Baleanu

**Affiliations:** 1 School of Mathematics and Statistics, Northwestern Polytechnical University, Xi’an, 710129 China; 2 Department of Mathematics, University of Management and Technology, Lahore, 54770 Pakistan; 3 School of Aerospace and Mechanical Engineering, Nanyang Technological University, Singapore, Singapore; 4 School of Energy and Power, Xi’an Jiaotong University, Xi’an, 7100049 China; 5 Department of Mathematics, Cankaya University, 06530 Balgat Ankara, Turkey; 6 Institute of Space Sciences, Magurele-Bucharest, Magurele-Bucharest, Romania; 7 Department of Medical Research, China Medical University Hospital, China Medical University, Taichung, Taiwan

**Keywords:** Engineering, Mathematics and computing

## Abstract

This study briefings the roles of Coriolis, and Lorentz forces on the dynamics of rotating nanofluids flow toward a continuously stretching sheet. The nanoparticles are incorporated because of their unusual qualities like upgrade the thermal transportation, which are very important in heat exchangers, modern nanotechnology, electronics, and material sciences. The primary goal of this study is to improve heat transportation. Appropriate similarity transformations are applied for the principal PDEs to transform into nonlinear dimensionless PDEs. A widely recognized Numerical scheme known as the Finite Element Method is employed to solve the resultant convective boundary layer balances. Higher input in the solvent fraction parameter has a rising effect on the primary velocity and secondary velocity magnitude, and decreasing impact on the distributions of temperature. It is seen that growing contributions of the Coriolis, and Lorentz forces cause to moderate the primary and secondary velocities, but the temperature and concentration functions show opposite trend. The concentration, temperature, and velocities distributions for suction case is prominently than that of injection case, but inverse trend is observed for local Nusselt and Sherwood numbers. These examinations are relevant to the field of plastic films, crystal growing, paper production, heat exchanger, and bio-medicine.

## Introduction

### Background study

The fluids which are engineered by the homogeneous dispersion of metal and metallic oxide particles at the nanoscale are known as nanofluids^1^. The theoretical and experimental investigations confirm from the literature that the existence of nanoparticles in host fluid greatly affects the thermophysical properties of base fluids which exhibits poor conductivity properties^2,3^. The convective nanofluid heat transfer flow attracts numerous researchers due to their fascinating applications in every field of Science and Engineering. To name a few, the diamond and ceramic nanoparticles are used to upsurge the dielectric properties of the mineral oil, the fluid with nanoparticles can be used for direct absorption of the sunlight in solar collectors, the Zink and Titanium Oxide particles have antibacterial behavior and therefore which can be used for biomedical applications such as drug delivery and cancer therapy, and many more^4–6^. Ali et al.^7^ discussed convective unsteady hybrid nanofluid flow within an upright channel by using Laplace Transform (LT) technique, in which authors developed fractional Maxwell fluid model is developed by using Caputo fractional differential operator. Awais et al.^8^ considered nanofluid assisting and opposing flow for exploring the Lie group analysis numerically with Adams-Bash forth technique (ABT). Abdelmalek et al.^9^ considered Cauchy’s stress tensor for third-grade fluid to investigate the dissipative nanofluid flow past an elongating sheet by adopting built-in Matlab routine bvp4c. Farooq et al.^10^ adopted a modified Cattaneo–Christov mass and heat flu model for the numerical exploration of the Carreau nanofluid flow by horizontal stretching cylinder subject to the convective conditions. Subba Rao et al.^11^ studied the nanofluid (Buongiorno model) flow above the isothermal upright cone. There are some articles related to boundary layer flow along a nanoparticles^12–14^. The non-Newtonian fluids are technological importance. In nature, Boger fluids are also non-Newtonian fluids^15,16^.

There have been rising fascination in the investigation of the impact of Lorentz force produced due to magnetic field because of its modern applications in fluid engineering, assembling of plastic materials, hot rolling, production of glass fiber, and medical treatment^17,18^. Magnetic doubly stratified mixed convective flow with heat generation/absorption effects reported by Abbasi et al.^19^ and Alhussain et al.^20^ securitized numerically on Cattaneo-Christov flux model for the magneto nanofluid flow past a spinning cone embedded in an anisotropic permeable medium in the presence of cross-diffusion (Soret and Dufour), Navier slip, and Stefan blowing effects. The insight into significance of particles aggregation and carbon nanotube subject to rotating environment studied by Acharya et al.^21,22^. Jawad et al.^23^ obtained approximate analytical solutions using HAM for the energy irreversibility analysis on hydromagnetic nanofluid flow past a nonlinear elongating permeable sheet. Jamshed^24^ employed Keller Box Method (KBM) to study the MHD nanofluid flow past a nonlinearly stretching sheet with entropy generation and viscous dissipation effects.

In the recent years, the growing interest in the examination of Coriolis force effects on the dynamics of fluid flow over a stretching sheet because of several real applications in astrophysical and geophysical problems, hot rolling, fiber production, and centrifugal bio-reactor^25^. Chu et al.^26^ explored numerically on nanofluid flow past a bidirectional periodically moving surface in the presence of nonlinear radiation and heat source/sink effects by employing (HAM) Homotopy Analysis Method. Ali et al.^18^ employed GFEM (Galerkin Finite Element Method) to explore the buoyant driven transient bio convective Maxwell nanofluid rotating 3D flow above the Riga plate with binary chemical reaction and activation energy. Ahmed et al.^27^ numerically investigated heat source/sink effects on stagnation point flow over stretching/shrinking rotating disk, Bilal et al.^28^ studied chemical reaction effects on magnetized nanofluid past a rotating cone with HAM, and khan et al.^29^ reported on the impact of heat source/sink effects subject to revised mass flu conditions for flow past rotating elongating cylinder. Ali et al.^30^ employed FEM to explore rotating Casson Carreau nanofluid flow over a sheet with Darcy–Forchheimer medium.

The scrupulous literature assessment cited above confirms that a little attention is made towards the Lorentz force embedded in Bogar nanofluid rotating flow past stretching surface under the influence of the Coriolis force and suction/injection, and present analysis is unique because of solvent fraction, Lorentz and Coriolis impact on dynamic of Bogar nanofluid. To the best of the author’s knowledge, all the cited reports are not deliberated on the elaborated problem. Therefore, the prime aim of the current investigation is to investigate the heat and mass transfer effects on transient hydromagnetic rotating nanofluid 3D flow with suction/injection effects. Very recently, the hydromagnetic boundary layer flow for both Newtonian and non-Newtonian nanofluid flow fields was investigated by numerous authors^31–33^ by employing GFEM with a weighted residual approach. The flow governing coupled highly nonlinear PDEs are solved by adopting a control volume approach by employing a Galerkin Finite Element Method^34,35^ with a weighted residual approach. The various flow field characteristics for numerous significant parameter changes are discussed and depicted pictorially. The computational results obtained through Matlab code blocks are corroborated with the existing literature and found to be a tolerable correlation. The friction factor, Nusselt number, and Sherwood number values are computed and documented in tabular form. This numerical study is relevant to the polymers, heat exchangers, bio-medicine, and astrophysical and geophysical problems.

### Research questions

This study provide answers to below related scientific research questions:

1. To observe the influence of Coriolis and Lorentz forces on the Boger fluid dynamics of temperature, fluid velocity, and concentration distributions with suction/injection?
2. What is the effect of combine affect of Brownian motion, thermophoresis, Coriolis and Lorentz forces on the mass transfer rate, skin friction tensors, and heat transfer rate with suction/injection?
3. Assess the Brownian motion, and thermophoresis parameters effect the temperature and tiny particles concentration profile?

## Geometry and mathematical formulation

**Figure 1:**
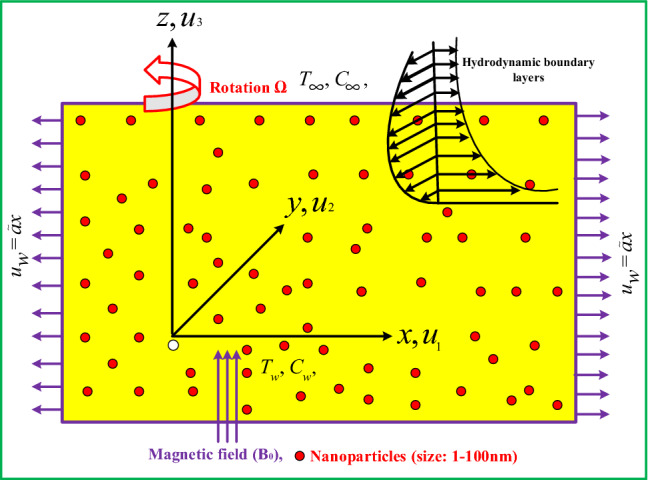
Physical configuration and coordinate system.

The transient magnetohydrodynamic 3*D* rotating flow of nanofluid past a bidirectional stretching sheet is considered. The physical flow configuration and coordinate system of elaborated problem is shown in Fig. 1, and flow is confined to $z\ge 0$. The nanofluid flow rotating about the z-direction with angular constant velocity $\Omega$. At $z = t = 0.0$, the surface is elongated in the direction of *x* along with velocity $u_w = {\tilde{a}}x$. In axial direction (z-direction), a static and uniform field of magnetic $B_0$ is applied. Less magnetic filed is induced due to low magnetic Reynolds number, so that Hall current and Ohmic dissipation are insignificant^30^. The ambient concentration and temperature is $C_\infty$ and $T_\infty$, while surface concentration and temperature is represented by $C_w$, and $T_w$, respectively. The field of velocity for the current elaborate problem is considered by $V = (u_1(x,y,z), u_2(x,y,z), u_3(x,y,z))$. In view of above assumptions and boundary layer approximation, the equations of conservation of mass, linear-momentums, temperature, and concentration are expressed as^36,37^:

1 $$\begin{aligned}{}&\partial _x{u}_1+\partial _y{u}_2+\partial _z{u}_3=0, \end{aligned}$$

2 $$\begin{aligned}{}&\rho _{nf}(\partial _t{u}_1+ {u}_1\partial _x{u}_1 + {u}_2\partial _y{u}_1 + {u}_3\partial _z{u}_1 + 2\Omega {u}_2)= -\partial _x p+\mu _{nf}\bigg (\frac{1+\beta _1}{1+\beta _2}\bigg )\partial _{zz}{u}_1 - \sigma _{n_f}B_0^2u_1, \end{aligned}$$

3 $$\begin{aligned}{}&\rho _{nf}(\partial _t{u}_2+ {u}_1\partial _x{u}_2 + {u}_2\partial _y{u}_2 + {u}_3\partial _z{u}_2 - 2\Omega {u}_1)= -\partial _y p+\mu _{nf}\bigg (\frac{1+\beta _1}{1+\beta _2}\bigg )\partial _{zz}{u}_2- \sigma _{n_f}B_0^2u_2, \end{aligned}$$

4 $$\begin{aligned}{}&\rho _{nf}(\partial _t{u}_3+ {u}_1\partial _x{u}_3 + {u}_2\partial _y{u}_3 + {u}_3\partial _z{u}_3)= -\partial _z p+\mu _{nf}\partial _{zz}{u}_3, \end{aligned}$$

5 $$\begin{aligned}{}&\partial _t{T} + {u}_1\partial _x{T} + {u}_2\partial _y{T} +{u}_3\partial _z{T} = \alpha _{n_f}\partial _{zz}{T} +{\tau }^*\left\{ D_b\partial _z{C}\partial _z{T} +\frac{D_T}{T_\infty }(\partial _z{T})^2\right\} , \end{aligned}$$

6 $$\begin{aligned}{}&\partial _t{C} + {u}_1\partial _x{C} + {u}_2\partial _y{C} +{u}_3\partial _z{C} = {D}_b\partial _{zz}{C} +\frac{D_T}{ T_\infty }\partial _{zz}{T}. \end{aligned}$$

In the above equations, *C* and *T*) are the tiny particles concentration, and temperature of fluid, ($D_T, D_b$) are the thermophoresis, and Brownian diffusion coefficients, $(\rho _{n_f}, \alpha _{n_f}, \mu _{n_f},\beta _1, \beta _2)$ are respectively the thermal diffusivity, density, dynamic viscosity of fluid, solvent fraction parameter, and ratio of relaxation time, respectively. The boundary conditions are^38,39^:

7 $$\begin{aligned}{}&t <0: {u}_1 = 0,\ {u}_2 = 0,\ {u}_3 = 0,\ {C} = {C}_\infty , \ {T} = {T}_\infty , \end{aligned}$$

8 $$\begin{aligned}{}&t\ge 0: {u}_1 ={a}(x),\ {u}_2 = 0,\ {u}_3 = -w_0,\ {C} = {C}_w, \ {T} = {T}_w,\ as\ z=0, \end{aligned}$$

9 $$\begin{aligned}{}&t\ge 0: {u}_1\rightarrow 0,\ {u}_2\rightarrow 0,\ {C}\rightarrow {C}_\infty ,\ {T}\rightarrow {T}_\infty ,\ as\ z \rightarrow \infty . \end{aligned}$$

The complexity of elaborated problem is reduced by introducing the below similarity transformations (see Refs.^36,38^):

10 $$\begin{aligned} \left. \begin{array}{rr} & u_1= {\tilde{a}}x\frac{\partial F(\zeta ,\eta )}{\partial \eta },\ u_2 = {\tilde{a}}xG(\zeta ,\eta ),\ u_3 = -\sqrt{{\tilde{a}}\nu \zeta }F(\zeta ,\eta ),\ \ \zeta = 1-e^{-\Gamma }, \eta = \sqrt{\frac{{\tilde{a}}xz^2}{\zeta \nu }},\,\\ \quad \quad \quad \quad \quad \\ & \quad \quad \quad \qquad \qquad \quad \Gamma = {\tilde{a}}t,\ T = (T_w-T_\infty )\theta (\zeta ,\eta )+T_\infty , \ C = (C_w-C_\infty )\Phi (\zeta ,\eta )+C_\infty . \end{array}\right\} \end{aligned}$$

In view of Eq. (10), the Eq. (1) is valid and the Eqs. (2–9) becomes below mention non-linear PDEs into transformed coordinate systems ($\zeta ,\eta$).

11 $$\begin{aligned}{}&\bigg (\frac{1+\beta _1}{1+\beta _2}\bigg )F'''+ 0.5\eta {F''}-0.5\zeta \eta {F''}+\zeta (FF''-F'^{2} - M^2F'+ 2\lambda {G})-\zeta (1-\zeta )\frac{\partial {F}'}{\partial \zeta }=0, \end{aligned}$$

12 $$\begin{aligned}{}&\bigg (\frac{1+\beta _1}{1+\beta _2}\bigg )G''+ 0.5\eta {G'}-0.5\zeta \eta {G'}+\zeta (FG'- 2\lambda {F}'- M^2G-F'G)-\zeta (1-\zeta )\frac{\partial {G}}{\partial \zeta }=0, \end{aligned}$$

13 $$\begin{aligned}{}&\theta '' + 0.5\eta (1-\zeta )Pr\theta ' +\zeta {Pr}F\theta ' +N_bPr\theta \Phi +N_tPr\theta '^2- \zeta (1-\zeta )Pr\frac{\partial \theta }{\partial \zeta }=0, \end{aligned}$$

14 $$\begin{aligned}{}&\Phi '' + 0.5\eta {Le}(1-\zeta )\Phi '+Le\zeta {F}\Phi ' + N_t{N^{-1}_b}\theta '' - \zeta (1-\zeta )Le\frac{\partial \Phi }{\partial \zeta }=0, \end{aligned}$$

15 $$\begin{aligned}{}&\left. \begin{array}{rr} &{}F(\zeta ,0) = \Gamma ,\ G(\zeta ,0) = 0,\ F'(\zeta ,0) = \Phi (\zeta ,0)=\theta (\zeta ,0) = 1,\ \zeta \ge = 0,\ at\ \eta =0 ,\\ &{}\quad \quad F'(\zeta ,\infty )\rightarrow 0,\ G(\zeta ,\infty )\rightarrow 0,\ \Phi (\zeta ,\infty )\rightarrow 0,\ \theta (\zeta ,\infty )\rightarrow 0,\ \zeta \ge 0,\ as\ \eta \rightarrow \infty , \end{array}\right\} \end{aligned}$$

where primes ($','','''$) denote the d.r.t $\eta$. The rotating $(\lambda )$, magnetic (*M*), Prandtl number (*Pr*), Lewis number (*Le*), Brownian motion $(N_b)$, thermophoresis $(N_t)$, and suction/injection ($\Gamma$) parameters are defined as:

$$\begin{aligned}{}&\lambda = \frac{\Omega }{{a}}, \ M = \sqrt{\frac{\sigma _{n_f}B_o^2}{\rho _f{\tilde{a}}}}, \ \ Pr = \frac{\nu }{\alpha _{n_f}}, \ Le = \frac{\nu }{{D}_B},\ Lb = \frac{\nu }{{D}_m},\ N_b = \tau \nu ^{-1}{D}_B ({C}_w -{C}_\infty ),\\&N_t = \frac{{D}_T (\tau {T}_w-\tau {T}_\infty )}{\nu {T}_\infty },\ \Gamma = \frac{w_0}{\sqrt{{\tilde{a}}\nu \zeta }}. \end{aligned}$$

Expressions of local Nusselt number, Sherwood number, and coefficients of skin friction are defined as:

16 $$\begin{aligned} Nu = \frac{xq_w}{\kappa ({T}_w-{T}_\infty )},\ Shr = \frac{xq_m}{{D}_B({C}_w-{C}_\infty )},\ C_{f_x} = \frac{\tau _w^x}{\rho {u}_1^2} ,\ C_{f_y} = \frac{\tau _w^y}{\rho {u}_1^2}. \end{aligned}$$

Here, tensor of skin friction at wall are $\tau _w^x = \mu \bigg (\frac{1+\beta _1}{1+\beta _2}\bigg )({\partial {u}_1}/{\partial z})_{z=0}$ (x-direction) and $\tau _w^y = \mu \bigg (\frac{1+\beta _1}{1+\beta _2}\bigg )({\partial {u}_2}/{\partial z})_{z=0}$ (y-direction), the mass, and heat flux at the sheet are $q_m = -{D}_B({\partial {C}}/{\partial z})_{z=0}$, and $q_w = -\kappa ({\partial {T}}/{\partial z})_{z=0}$. Using Eq. (10):

17 $$\begin{aligned} \left\{ \begin{array}{ll} & C_{f_x}{Re_x}^{1/2} = \bigg (\frac{1+\beta _1}{1+\beta _2}\bigg )\frac{{F}''(0)}{\sqrt{\zeta }}, C_{f_y}{Re_x}^{1/2} = \bigg (\frac{1+\beta _1}{1+\beta _2}\bigg )\frac{{G}'(0)}{\sqrt{\zeta }},\\ & Nu_x{Re_x}^{1/2} = -\frac{\left[ \theta '(0)\right] }{\sqrt{\zeta }}, Shr_x{Re_x}^{1/2} = - \frac{\left[ \Phi '(0)\right] }{\sqrt{\zeta }}. \end{array}\right. \end{aligned}$$

## Numerical solution

### Finite element method

This numerical technique is very powerful tool reported by the experts to solve the modern engineering and industrial problems due to its accuracy and computability^18,40^. In this technique, below steps are involved:

### Finite-element discretisation

First, the whole computational domain is distributed into sub-interval of a finite number and these sub-intervals are called elements, and set of these elements represent the finite element mesh.

### Generation of the element equations

(i) On the base of finite element mesh, a variational formulation of the problem is performed.
(ii) In above (i), the element equations are generated with the aid of approximation solution.
(iii) Construct a stiffness matrix by using interpolation functions.

### Assembly of element equations

After generation of the elements equation, we assembled all the generated elements equations through inter element continuity condition, and obtained a model which called global finite element model over a entire computational domain.

### Imposition of boundary conditions

The elaborated boundary conditions of the current problem are utilized on the obtained assembled equations.

### Solution of assembled equations

Finally, we solved the assembled equations iteratively. To justify the validity of finite element computations, a study for grid independence is conducted. The whole domain is divided into different mesh density of grids size, and observe no more variation after $100\times 100$, so we fixed the all computations on $100\times 100$ grid size (see Table 1). The comparison with earlier studies are documented in Tables 2 and 3 in particular cases are carried out to get the conformism of the precision of the solution methodology. It is witnessed that the current numerical computations are in close agreement with the existing literature in some limiting cases. The Finite Element computations for friction factor along with axial and transverse directions $-F''(0) \& -G(0)$ respectively tabulated in Table 2 for different values of rotation parameter $\lambda = 0, 1, 2, 5$ at $\zeta = 1$. It is observed from Table 2 that the numerical results so obtained are in good correlation with the results reported by Ali et al.^41^, and Wang^42^. Further, in Table 3, the Nusselt number $-\theta (0)$ values are corroborated between Ali et al.^43^ and Shafique et al.^44^, and present FEM results for diverse values $\lambda , \beta , \& Pr$, and found that they are in good agreement. Hence, the confidence in the numerical computations and confirm that the Finite Element Computations obtained through Matlab code are attained the good convergence rate.

**Table 1:** Study of Grid independence for different grid sizes at $\zeta = 1.0$.

Grid size	$-F''(\zeta ,0)$	$-G'(\zeta ,0)$	$-\theta '(\zeta ,0)$	$-\Phi '(\zeta ,0)$
10 $\times$ 10	2.7911	1.9890	0.7145	3.2679
40 $\times$ 40	2.7611	1.0827	0.6711	3.1553
75 $\times$ 75	2.7553	1.0747	0.6865	3.0912
100 $\times$ 100	2.7550	1.0743	0.6861	3.0908
120 $\times$ 120	2.7549	1.0740	0.6860	3.0905

**Table 2:** Comparison of skin friction $-G'(0)$ and $-F''(0)$ for various values of $\lambda$ at $\zeta =1$ when ignore other involved parameters.

$\lambda$	Ali et al.^41^		Wang^42^		Present	
	$-F''(0)$	$-G'(0)$	$-F''(0)$	$-G'(0)$	$-F''(0)$	$-G'(0)$
0.0	1.00000	0.00000	1.0000	0.0000	1.00000	0.00000
1.0	1.32501	0.83715	1.3250	0.8371	1.32501	0.83715
2.0	1.65232	1.28732	1.6523	1.2873	1.65232	1.28732
5.0	2.39026	2.15024	–	–	2.39026	2.15024

**Table 3:** Comparison of Nusselt number $-\theta '(0)$ for $\zeta =1$ and different values of rotating parameters $\lambda$ when other parameters are zeros.

$\lambda$	$\beta$	Pr	Ali et al.^43^	Shafique et al.^44^	FEM (our results)
0.2	0.2	1.0	0.546683	0.54670	0.5466828
–	0.4	–	0.528090	0.52809	0.5280903
–	0.6	–	–	0.51009	0.5100870
–	0.8	–	0.492547	0.49255	0.4925468

To solve the Eqs. (11) to (14) along boundary conditions (15), first consider:

18 $$\begin{aligned} F'= H, \end{aligned}$$

The set of Eqs. (11)–(15) reduced to lesser order:

19 $$\begin{aligned}{}&\bigg (\frac{1+\beta _1}{1+\beta _2}\bigg )H''+0.5(1-\zeta )\eta {H}'+\zeta ({F}{H}'-{H}^2+2\lambda {G}-M^2H) = \zeta \frac{\partial {H}}{\partial \zeta }-\zeta ^2\frac{\partial {H}}{\partial \zeta }, \end{aligned}$$

20 $$\begin{aligned}{}&\bigg (\frac{1+\beta _1}{1+\beta _2}\bigg )G''+0.5\eta {G}'-0.5\eta \zeta {G}'+\zeta (FG'- 2\lambda {F}'- M^2G-F'G) = \zeta \frac{\partial {G}}{\partial \zeta }-\zeta ^2\frac{\partial {G}}{\partial \zeta }, \end{aligned}$$

21 $$\begin{aligned}{}&Pr^{-1}\theta ''+ 0.5\eta (1-\zeta )\theta '+\zeta {F}\theta '+ N_b\theta '\Phi ' + N_t\theta '^2 = \zeta \frac{\partial {\theta }}{\partial \zeta }-\zeta ^2\frac{\partial {\theta }}{\partial \zeta }, \end{aligned}$$

22 $$\begin{aligned}{}&\Phi ''+ 0.5Le(1-\zeta )\eta \Phi '+Le\zeta {F}\Phi '+ {N_t}{N^{-1}_b}\theta ''^2 = \zeta (1-\zeta )Le\frac{\partial {\Phi }}{\partial \zeta }, \end{aligned}$$

23 $$\begin{aligned}{}&\left. \begin{array}{rr} &{}F(\zeta ,0)=\Gamma ,\ G(\zeta ,0)=0,\ \Phi (\zeta ,0)=H(\zeta ,0)=\theta (\zeta ,0) = 1,\ \zeta \ge = 0,\ at\ \eta =0 ,\\ &{}\quad \quad H(\zeta ,\infty )\rightarrow 0,\ G(\zeta ,\infty )\rightarrow 0,\ \Phi (\zeta ,\infty )\rightarrow 0,\ \theta (\zeta ,\infty )\rightarrow 0,\ \zeta \ge 0,\ as\ \eta \rightarrow \infty . \end{array}\right\} \end{aligned}$$

For numerical computation, the plate length fixed at $\zeta = 1.0$ and thickness at $\eta = 5.0$. The variational form connected along with Eqs. (18)–(22) can be written as:

24 $$\begin{aligned}{}&\int \limits _{\Omega _e}w_{f_1}\{F'-H\}d\Omega _e = 0, \end{aligned}$$

25 $$\begin{aligned}{}&\int \limits _{\Omega _e}w_{f_2}\bigg \{\bigg (\frac{1+\beta _1}{1+\beta _2}\bigg )H''+\frac{1}{2}(1-\zeta )\eta {H}'+\zeta ({F}{H}'-{H}^2+2\lambda {H}-M^2H) - \zeta (1-\zeta )\frac{\partial {H}}{\partial \zeta }\bigg \}d\Omega _e= 0, \end{aligned}$$

26 $$\begin{aligned}{}&\int \limits _{\Omega _e}w_{f_3}\bigg \{\bigg (\frac{1+\beta _1}{1+\beta _2}\bigg )G''+\frac{1}{2}(1-\zeta )\eta {G}'+\zeta ({F}{G}' -{H}{G}-2\lambda {H})-\zeta (1-\zeta )\frac{\partial {G}}{\partial \zeta }\bigg \}d\Omega _e = 0, \end{aligned}$$

27 $$\begin{aligned}{}&\int \limits _{\Omega _e}w_{f_4}\bigg \{\theta ''+ \frac{Pr}{2}(1-\zeta )\eta \theta '+Pr\zeta {F}\theta '+ N_bPr\theta '\Phi ' + N_tPr(\theta ')^2 - Pr\zeta (1-\zeta )\frac{\partial \theta }{\partial \zeta }\bigg \}d\Omega _e = 0, \end{aligned}$$

28 $$\begin{aligned}{}&\int \limits _{\Omega _e}w_{f_5}\bigg \{\Phi ''+ 0.5Le\eta (1-\zeta )\Phi '+\zeta {LeF}\Phi '+ \frac{N_t}{N_b}(\theta '')^2 -\zeta (1-\zeta )Le\frac{\partial \Phi }{\partial \zeta }\bigg \}d\Omega _e = 0. \end{aligned}$$

**Figure 2:**
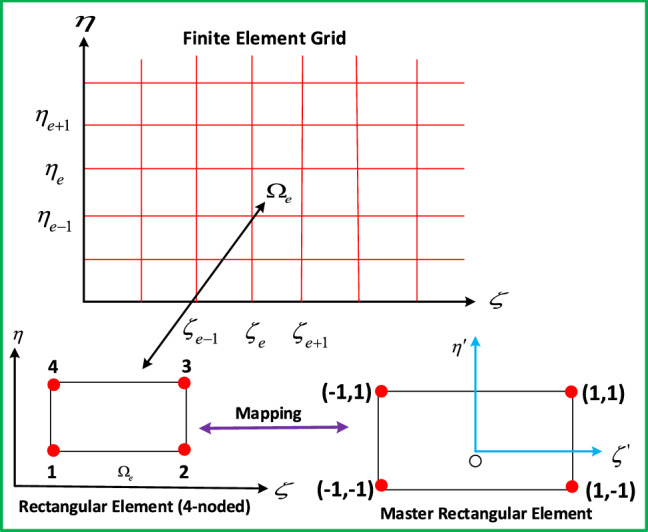
Rectangular grid.

Here $w_{f_1}$, $w_{f_2}$, $w_{f_3}$, $w_{f_4}$ for trial functions. Divide the domain ($\Omega _e$) into 4-nodded elements (see Fig. 2). The related approximations of finite element are:

29 $$\begin{aligned} F = \sum _{j=1}^4 F_j \Upsilon _j(\zeta ,\eta ),\ H = \sum _{j=1}^4 H_j \Upsilon _j(\zeta ,\eta ),\ G = \sum _{j=1}^4 G_j \Upsilon _j(\zeta ,\eta ),\ \theta = \sum _{j=1}^4 \theta _j \Upsilon _j(\zeta ,\eta ),\ \Phi = \sum _{j=1}^4\Phi _j\Upsilon _j(\zeta ,\eta ). \end{aligned}$$

Here, $\Upsilon _1$, $\Upsilon _2$, $\Upsilon _3$, and $\Upsilon _4$ are the linear interpolation functions for $\Omega _e$ are given by:

30 $$\begin{aligned} \left. \begin{array}{ll} &{}\Upsilon _1 = \frac{(\zeta _{e+1}-\zeta )(\eta _{e+1}-\eta )}{(\zeta _{e+1}-\zeta _e)(\eta _{e+1}-\eta _e)},\ \Upsilon _2 = \frac{(\zeta -\zeta _e)(\eta _{e+1}-\eta )}{(\zeta _{e+1}-\zeta _e)(\eta _{e+1}-\eta _e)},\\ &{}\Upsilon _3 = \frac{(\zeta -\zeta _e)(\eta -\eta _e)}{(\zeta _{e+1}-\zeta _e)(\eta _{e+1}-\eta _e)},\ \Upsilon _4 = \frac{(\zeta _{e+1}-\zeta )(\eta -\eta _e)}{(\zeta _{e+1}-\zeta _e)(\eta _{e+1}-\eta _e)}. \end{array}\right\} \end{aligned}$$

The develop finite-elements model of the equations is below:

31 $$\begin{aligned} \begin{bmatrix} [L^{11}] &{} [L^{12}] &{} [L^{13}] &{} [L^{14}] &{} [L^{15}]\\ [L^{21}] &{} [L^{22}] &{} [L^{23}] &{} [L^{24}] &{} [L^{25}]\\ [L^{31}] &{} [L^{32}] &{} [L^{33}] &{}[ L^{34}] &{} [L^{35}] \\ [L^{41}] &{} [L^{42}] &{} [L^{43}] &{} [L^{44}] &{} [L^{45}] \\ [L^{51}] &{} [L^{52}] &{} [L^{53}] &{} [L^{54}] &{} [L^{55}] \end{bmatrix} \begin{bmatrix} \{F\}\\ \{Q\}\\ \{H\} \\ \{\theta \}\\ \{\Phi \} \end{bmatrix} = \begin{bmatrix} \{R_1\} \\ \{R_2\}\\ \{R_3\} \\ \{R_4\}\\ \{R_5\} \end{bmatrix} \end{aligned}$$

where $[L^{mn}]$ and $[R_m]$ (m,n=1,2,3,4) are defined as:

$$\begin{aligned} L^{11}_{ij}= & {} \int \limits _{\Omega _e} \Upsilon _i\frac{d\Upsilon _j}{d\eta }d\Omega _e, L^{12}_{ij} = -\int \limits _{\Omega _e} \Upsilon _i\Upsilon _jd\Omega _e, L^{13}_{ij} = L^{14}_{ij} = L^{15}_{ij} = L^{21}_{ij} = L^{24}_{ij} = L^{25}_{ij} = 0, \\ L^{22}_{ij}= & {} -\bigg (\frac{1+\beta _1}{1+\beta _2}\bigg )\int \limits _{\Omega _e} \frac{d\Upsilon _i}{d\eta } \frac{d\Upsilon _j}{d\eta }d\Omega _e +\frac{1}{2}(1-\zeta )\eta \int \limits _{\Omega _e}\Upsilon _i \frac{d\Upsilon _j}{d\eta }d\Omega _e+\zeta \int \limits _{\Omega _e} {\bar{F}}\Upsilon _i\frac{d\Upsilon _j}{d\eta }d\Omega _e-\zeta \int \limits _{\Omega _e}{\bar{H}}\Upsilon _i \Upsilon _j d\Omega _e -\zeta (1-\zeta )\\& \times \int \limits _{\Omega _e}\Upsilon _i \frac{d\Upsilon _j}{d\zeta }d\Omega _e - M^2\zeta \int \limits _{\Omega _e}\Upsilon _i \Upsilon _j d\Omega _e,\ L^{23}_{ij} = 2\lambda \zeta \int \limits _{\Omega _e}\Upsilon _i \Upsilon _j d\Omega _e, L^{31}_{ij}=L^{34}_{ij}=L^{35}_{ij} =0,\\ L^{32}_{ij} = \; & 2\lambda \zeta \int \limits _{\Omega _e}\Upsilon _i \Upsilon _j d\Omega _e, L^{41}_{ij}=L^{42}_{ij}=L^{43}_{ij}=0, L^{33}_{ij}= -\bigg (\frac{1+\beta _1}{1+\beta _2}\bigg )\int \limits _{\Omega _e}\frac{d\Upsilon _i}{d\eta }\frac{d\Upsilon _j}{d\eta }d\Omega _e\nonumber \\&+ \frac{1}{2}(1-\zeta )\eta \int \limits _{\Omega _e}\Upsilon _i \frac{d\Upsilon _j}{d\eta }d\Omega _e+\zeta \int \limits _{\Omega _e} {\bar{F}}\Upsilon _i\frac{d\Upsilon _j}{d\eta }d\Omega _e -\zeta \int \limits _{\Omega _e}{\bar{H}}\Upsilon _i \Upsilon _j d\Omega _e-\zeta (1-\zeta )\int \limits _{\Omega _e}\Upsilon _i \frac{d\Upsilon _j}{d\zeta }d\Omega _e,\\ L^{44}_{ij}= & {} -\int \limits _{\Omega _e} \frac{d\Upsilon _i}{d\eta } \frac{d\Upsilon _j}{d\eta }d\Omega _e + \frac{Pr}{2}(1-\zeta )\eta \int \limits _{\Omega _e}\Upsilon _i \frac{d\Upsilon _j}{d\eta }d\Omega _e +Pr\zeta \int \limits _{\Omega _e} {\bar{F}}\Upsilon _i\frac{d\Upsilon _j}{d\eta }d\Omega _e +PrN_b\int \limits _{\Omega _e}{\bar{\Phi }}'\Upsilon _i \frac{d\Upsilon _j}{d\eta }d\Omega _e\\&+PrN_t\int \limits _{\Omega _e}{\bar{\theta }}'\Upsilon _i \frac{d\Upsilon _j}{d\eta }d\Omega _e -Pr\zeta (1-\zeta )\int \limits _{\Omega _e}\Upsilon _i \frac{d\Upsilon _j}{d\zeta }d\Omega _e, L^{45}_{ij}=L^{51}_{ij} =L^{52}_{ij}=L^{53}_{ij} = 0,\\ L^{54}_{ij}= & {} -\frac{N_t}{N_b}\int \limits _{\Omega _e}\frac{d\Upsilon _i}{d\eta }\frac{d\Upsilon _j}{d\eta }d\Omega _e, L^{55}_{ij}= -\int \limits _{\Omega _e} \frac{d\Upsilon _i}{d\eta } \frac{d\Upsilon _j}{d\eta }d\Omega _e + \frac{Le}{2}(1-\zeta )\eta \int \limits _{\Omega _e}\Upsilon _i \frac{d\Upsilon _j}{d\eta }d\Omega _e +Le\zeta \int \limits _{\Omega _e} {\bar{F}}\Upsilon _i\frac{d\Upsilon _j}{d\eta }d\Omega _e\\&- Le\zeta (1-\zeta )\int \limits _{\Omega _e}\Upsilon _i \frac{d\Upsilon _j}{d\zeta }d\Omega _e, \end{aligned}$$

and

32 $$\begin{aligned}{}&R^1_i = \Gamma , \ R^2_i = -\oint \limits _{\Gamma _e} \Upsilon _i n_{\eta }\frac{\partial {H}}{\partial \eta } \,ds, \ R^3_i = -\oint \limits _{\Gamma _e} \Upsilon _i n_{\eta }\frac{\partial {G}}{\partial \eta } \,ds, \ R^4_i = -\oint \limits _{\Gamma _e} \Upsilon _i n_{\eta }\frac{\partial \theta }{\partial \eta }\,ds,\nonumber \\&R^5_i = - \oint \limits _{\Gamma _e} \Upsilon _i n_{\eta }\frac{\partial \Phi }{\partial \eta }\,ds - \frac{Nt}{Nb}\oint \limits _{\Gamma _e}\Upsilon _i n_{\eta }\frac{\partial \theta }{\partial \eta } \,ds, \end{aligned}$$

where, ${{\bar{F}}} = \sum _{j=1}^4 {{\bar{F}}}_j \Upsilon _j$, ${{\bar{H}}} = \sum _{j=1}^4 {{\bar{H}}}_j \Upsilon _j$, ${{\bar{G}}} = \sum _{j=1}^4 \bar{G}_j\Upsilon _j$, ${{\bar{\theta }}}' = \sum _{j=1}^4 \bar{\theta }'_j\Upsilon _j$, and ${{\bar{\Phi }}}'= \sum _{j=1}^4 \bar{\Phi }'_j\Upsilon _j$ supposed to be the known values. Evaluate 6 functions at each node, acquired 61206 equations after assembly are nonlinear, so linearize through an iterative scheme with $10^{-5}$ required precision.

## Results and discussion

In this section, we show the significance of suction/injection on dynamics of Maxwell rotating fluid subject to Coriolis force, magnetohydrodynamic, and gyrotactic microorganism via finite element simulation. In each of the figures for these quantities, three sets of curves are drawn for varying values of suction/injection $(\Gamma )$ parameter: (i) $\Gamma = -0.2$ (suction), (ii) $\Gamma = 0.0$ (static), and (iii) $\Gamma = 0.2$ (for injection and Table 1). The default values of the parameters in the study were: $\lambda = M = 1.0$, $N_b = N_t = 0.2$, $\beta _1 = 0.2$, $\beta _2 = 2.0$, $Le = 10$, $Pr = 6.2$.

**Figure 3:**
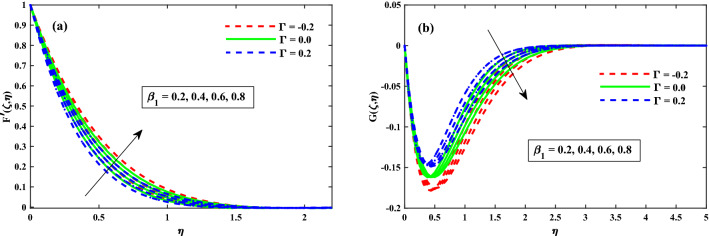
Impact of $\beta _1$ on $F'(\zeta ,\eta )$ in x-direction, and $G(\zeta ,\eta )$ in y-direction at $\zeta =1$.

**Figure 4:**
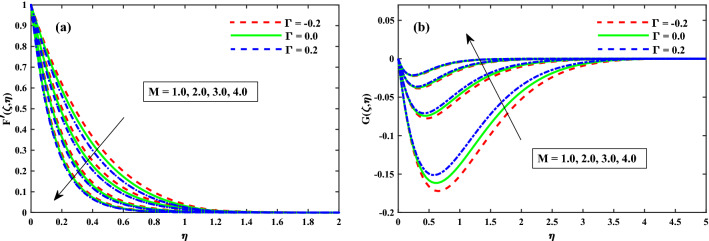
Impact of *M* on $F'(\zeta ,\eta )$ in x-direction, and $G(\zeta ,\eta )$ in y-direction at $\zeta =1$.

**Figure 5:**
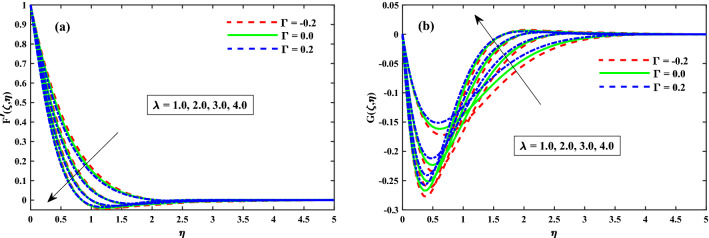
Impact of $\lambda$ on $F'(\zeta ,\eta )$ in x-direction, and $G(\zeta ,\eta )$ in y-direction at $\zeta =1$.

**Figure 6:**
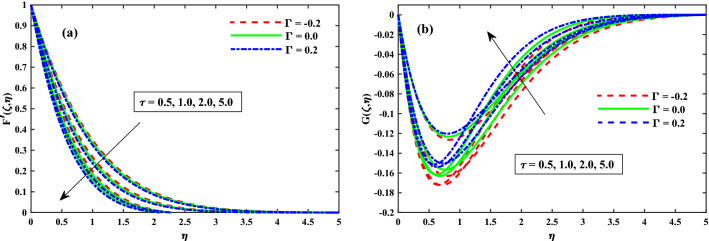
Impact of $\tau$ on $F'(\zeta ,\eta )$ in x-direction, and $G(\zeta ,\eta )$ in y-direction at $\zeta =1$.

The primary and secondary velocity distributions for different values of solvent fraction parameter, magnetic field parameter, rotation parameter, and unsteady parameter are portrayed in Figs. 3, 4, 5 and 6, respectively. The velocity distributions for various parameters is portrayed in Fig. 3a,b and figure exhibits that the velocity distributions is upgraded by solvent fraction parameter $\beta _1$. Actually, at low Reynolds number, the flow rate of Bogar nanofluid increases so at constant viscosity, Bogar fluid is highly elastic. Figure 4a,b displays that $F'(\zeta ,\eta )$ and $G'(\zeta ,\eta )$ for different values of magnetic field parameter. The existence of the resistive force in the form of Lorentz force is due to the inclusion of enhancing external magnetic field and leads to deceleration of the axial momentum in Fig. 4a while an opposite behavior is perceived for transverse momentum in Fig. 4b. The axial momentum $F'(\zeta ,\eta )$ and Transverse momentum $G'(\zeta ,\eta )$ for several values of rotation parameter portrayed in Fig. 5a,b respectively. It is witnessed from Fig. 5a that the declining of the axial momentum for growing values of rotation parameter due to Coriolis force whereas an opposed action is alleged for transverse momentum in Fig. 5b. For growing values of time-dependent parameter, the increase in the thickness of momentum boundary layer in the transverse direction and reduction of the thickness of the momentum boundary layer in the axial direction is depicted in Fig. 6a,b respectively. Thus, it confirms that the unsteadiness parameter is vital in controlling the momentum in the axial direction. Moreover from these graphs, it is perceived that the $F'(\zeta ,\eta )$ reduces against the strength of $\Gamma = 0.2$ (injection), but it is slightly boosted up directly with $\Gamma = -0.2$ (suction) parameter.

**Figure 7:**
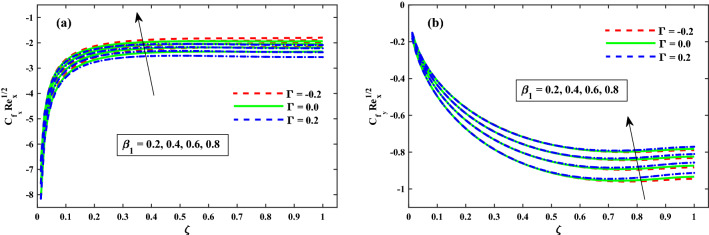
Impact of $\beta _1$ on $Cf_xRe^{1/2}_x$ in x-direction, and $Cf_yRe^{1/2}_y$ in y-direction.

The sketches of $C_{f_x}{Re_x}^{1/2}$ (friction factor) along axial and $C_{f_y}{Re_x}^{1/2}$ (transverse directions) near the surface for the range of $\zeta (0:0.2:1)$ is represented in Fig. 7a,b for $\beta _1(0.2:0.2:0.8)$. Figure 7a shows that for increasing $\zeta (0\rightarrow 1$, the distribution $(C_{f_x}{Re_x}^{1/2})$ is increased gradually up to a constant rate and then after no significant change is observed. Whereas for enhancing $\beta _1$, a significant reduction in $(C_{f_x}{Re_x}^{1/2})$ near the sheet surface is observed. For increasing $\zeta (0\rightarrow 1$, the distribution of $(C_{f_y}{Re_x}^{1/2})$ is reduced gradually up to a constant rate and then after no significant change is observed as shown Fig. 7b, while enhancing $\beta _1$. The significant difference in the values $(C_{f_y}{Re_x}^{1/2})$ near the sheet surface is observed. Moreover from these graphs, it is perceived that the values of $(C_{f_x}{Re_x}^{1/2})$ and $(C_{f_y}{Re_x}^{1/2})$ for the case of $\Gamma = 0.2$ (injection) are lesser than the case of $\Gamma = -0.2$ (suction).

**Figure 8:**
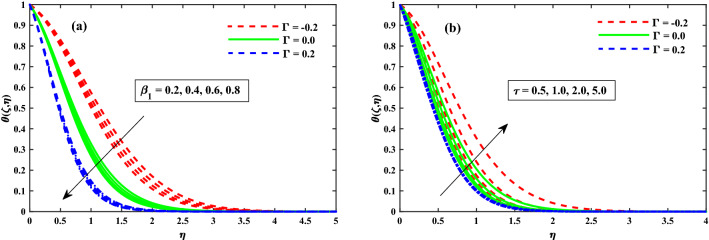
Impact of $\beta _1$ and $\tau$ on $\theta (\zeta ,\eta )$ at $\zeta =1$.

**Figure 9:**
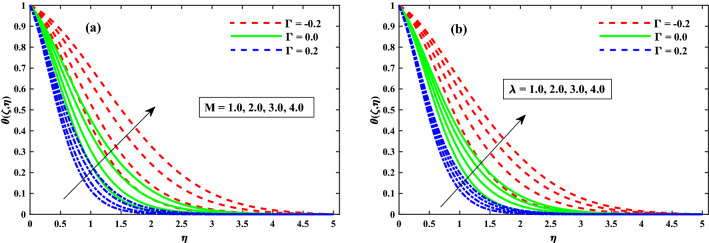
Impact of *M* and $\lambda$ on $\theta (\zeta ,\eta )$ at $\zeta =1$.

**Figure 10:**
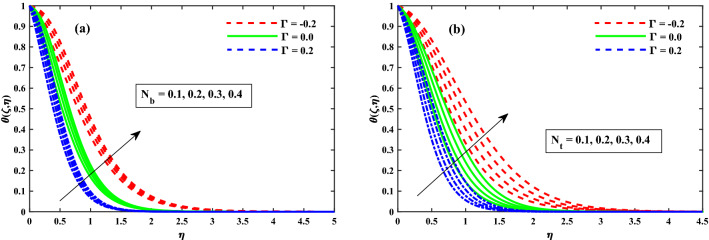
Impact of $N_b$ and $N_t$ on $\theta (\zeta ,\eta )$ at $\zeta =1$.

**Figure 11:**
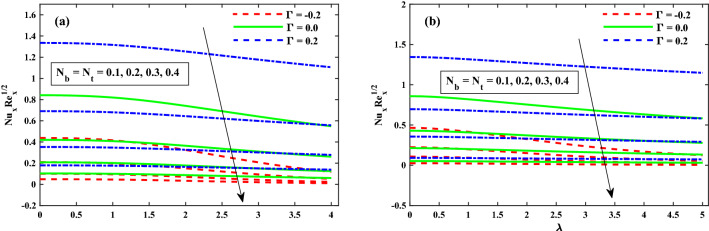
Fluctuation of $Nu_xRe_x^1/2$ against $N_b$, $N_t$, *M*, and $\lambda$.

The $\theta (\zeta ,\eta )$ distribution for various parameters is portrayed in Figs. 8, 9 and 10. The influence of effect of solvent fraction parameter $(\beta _1)$ and time-dependent $(\tau )$ on temperature is shown in Fig. 8a,b, respectively. The $\theta (\zeta ,\eta )$ profiles is decreased along with $\beta _1$, and increased against growing strength of unsteady parameter. Figure 9 exhibits that the temperature distributions is upgraded by magnetic field parameter. The net resulting net force usually known as resistive Lorentz force between the external magnetic field and internal electric field controls the flow momentum, which is depicted in Fig. 9a, while the thickness of the thermal boundary layer is increased for enhancing $\lambda$ as shown in Fig. 9b. The impact of thermophoresis $(N_t)$ and Brownian motion $(N_b)$ parameters on temperature is shown in Fig. 10a,b respectively. The distribution of temperature profiles is increased along with $N_b$ and $N_t$. Further, from these graphs, it is perceived that the $\theta (\zeta ,\eta )$ reduces against the strength of $\Gamma = 0.2$ (injection), but it is boosted up directly with $\Gamma = -0.2$ (suction) parameter. The sketches of local Nusselt number $(Nu_{x}{Re_x}^{1/2})$ at $Nt \& Nb(0.1:0.1:0.3)$ is represented in Fig. 11a,b for $M(0:1:5) \& \lambda (0:1:5)$ respectively. For increasing *M* and $\lambda$, the distribution of $(Nu_{x}{Re_x}^{1/2})$ is decreased gradually. A significant reduction in $(Nu_{x}{Re_x}^{1/2})$ near the sheet surface is observed for growing values of $Nt \& Nb$. It is also perceived from the figure that the for the case of $\Gamma = 0.2$ (injection) has a larger amount in $(Nu_{x}{Re_x}^{1/2})$.

**Figure 12:**
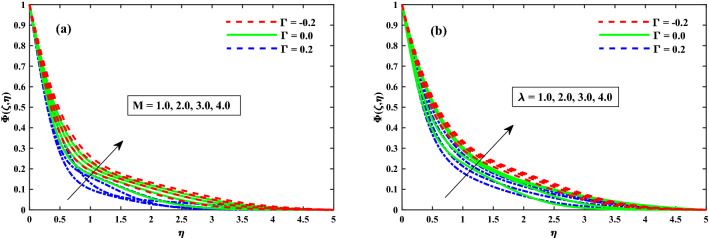
Impact of *M* and $\lambda$ on $\Phi (\zeta ,\eta )$ at $\zeta =1$.

**Figure 13:**
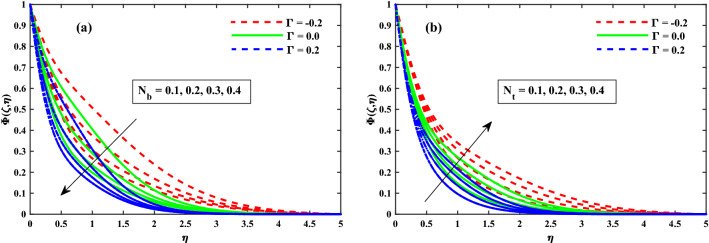
Impact of $N_b$ and $N_t$ on $\Phi (\zeta ,\eta )$ at $\zeta =1$.

**Figure 14:**
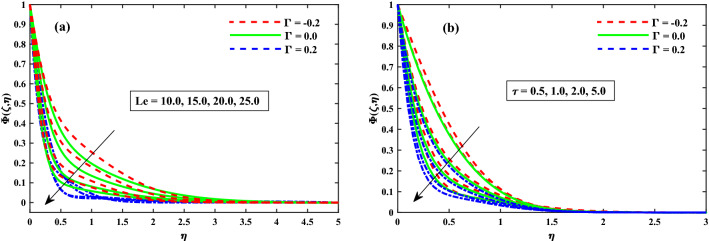
Impact of *Le* and $\tau$ on $\Phi (\zeta ,\eta )$ at $\zeta =1$.

**Figure 15:**
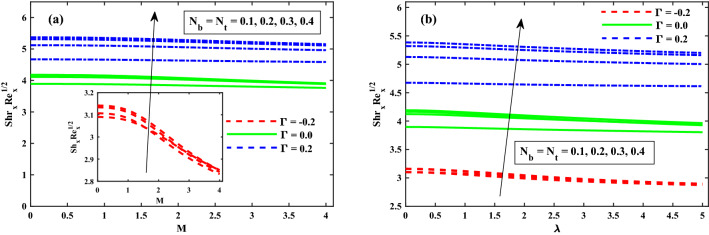
Fluctuation of $Shr_xRe_x^1/2$ against $N_b$, $N_t$, *M*, and $\lambda$.

Figure 12a,b displays that $(\Phi (\zeta ,\eta ))$ for different values of magnetic field *M*, and rotation $\lambda$ parameters. The concentration profiles are enhanced for increasing values of the magnetic field and rotating parameters as shown in Fig. 12a,b. From Fig. 13a,b revealed that the $\Phi (\zeta ,\eta )$ distribution profile is decrease again growing input of $N_b$, and it notably rise against the $N_t$. It is reported notably fall against the Lewis number *Le*, and time dependent parameter $(\tau )$ parameters (see Fig. 14a,b). The behavior of local Sherwood number $(Shr_{x}{Re_x}^{1/2})$ is portrayed in Fig. 15a,b for $M(0:1:5) \& \lambda (0:1:5)$ respectively at $Nt \& Nb(0.1:0.1:0.3)$. For increasing *M* and $\lambda$, the distribution of $(Shr_{x}{Re_x}^{1/2})$ is decreased. Whereas for increasing $Nt \& Nb$, an opposite trend is observed and it is also witnessed that the $\Gamma = 0.2$ (injection) case has larger $(Shr_{x}{Re_x}^{1/2})$ than that of $\Gamma = -0.2$ (suction) case.

## Conclusions

In this report, the finite element analysis on the transient magnetohydrodynamic three-dimensional rotating flow of nanofluid flow past a bidirectional stretching sheet with suction/injection, Coriolis, and Lorentz forces has been explored numerically. Based on the observed analysis of outcomes, it is worth concluding that:

1. Higher input in the Coriolis and Lorentz has a decreasing effect on the primary velocity and secondary velocity magnitude, and
  an increasing impact on the distributions of temperature and concentration.
  increase the magnitude of $Cf_xRe_x^{1/2}$ (skin friction factor).
  the velocity, temperature, and concentrations components reduce against the strength of injection.
2. Higher input in the solvent fraction parameter has a rising effect on the primary velocity and secondary velocity magnitude, and
  decreasing impact on the distributions of temperature.
  decrease the magnitude of $Cf_xRe_x^{1/2}$ (skin friction factor).
3. Simultaneous increase in Brownian motion and thermophoresis parameters have an increasing impact on the distribution of temperature, and
  a negative effects on $Nu_{x}{Re_x}^{1/2}$ and positive effects on $Shr_{x}{Re_x}^{1/2}$.
  injection has a larger amount in $Nu_{x}{Re_x}^{1/2}$.
  injection case has larger $Shr_{x}{Re_x}^{1/2}$ and $Re_x^{1/}N_x$ than that of suction case.
4. Tiny particles concentration diminishes against incremented Lewis number, unsteady, and Brownian motion parameters and exhibits rise against thermophoresis.

After successful computational struggle of parametric influence on the fluid dynamics, this study can be extend in future for Maxwell-nanofluid, Oldroyd-B nanofluid, comparative study between tangent-hyperbolic and Maxwell nanofluid, and viscoelastic Jeffrey’s nanofluids.
